# Prognostic Role of Serum Wisteria Floribunda Agglutinin-Positive Mac-2 Binding Protein Level in Early Stage Hepatocellular Carcinoma

**DOI:** 10.1038/s41598-020-62631-6

**Published:** 2020-03-27

**Authors:** Joseph Lin, Chih-Jan Ko, Yu-Ju Hung, Ping-Yi Lin, Kuo-Hua Lin, Chia-En Hsieh, Chen-Te Chou, Yao-Li Chen

**Affiliations:** 1 Department of General Surgery, Changhua Christian Hospital, Changhua, Taiwan; 2 School of Medicine, Kaohsiung Medical University, Kaohsiung, Taiwan; 3 Transplant Medicine & Surgery Research Centre, Changhua Christian Hospital, Changhua, Taiwan; 4 Department of Molecular Biotechnology, College of Biotechnology and Bioresources, Dayeh University, Changhua City, Taiwan; 5 Department of Radiology, Changhua Christian Hospital, Changhua City, Taiwan; 6 College of Nursing and Health Sciences, Dayeh University, Changhua City, Taiwan

**Keywords:** Cancer, Biomarkers, Gastroenterology

## Abstract

The purpose of this study is to evaluate the prognostic value of preoperative Wisteria floribunda agglutinin-positive Mac-2 binding protein (WFA+-M2BP) in predicting overall survival for patients with hepatitis B- and hepatitis C-related early-stage hepatocellular carcinoma (ESHCC) after liver resection. Post-operative survival rates were compared according to WFA+-M2BP level and tumor stage. Six hundred and ten patients were identified and 198 were removed after application of the exclusion criteria; the median follow-up time was 4.33 years, and cancer-related death occurred in 117 (28.4%) patients. Age (p = 0.03), fibrosis grade (p = 0.042), cancer stage (p = 0.01), and WFA+-M2BP level (p = 0.001) were identified as independent risk factors for poor overall survival. The overall survival rates at 3 and 5 years for patients with WFA+-M2BP ≤ 1.12 were 0.92 and 0.90, respectively, and 0.76 and 0.61 for patients with WFA+-M2BP > 1.12 (p < 0.001). During the analysis of survival prediction, serum WFA+-M2BP level exhibited a higher log-likelihood and a lower AIC value compared to TNM stage (log likelihood: −638; AIC: 1279). Pre-operative serum WFA+-M2BP level provided important prognostic information after curative hepatic resection in our study.

## Introduction

Hepatocellular carcinoma (HCC) is one of the most common cancers worldwide with high prevalence in the Asia Pacific region where hepatitis B virus (HBV) and hepatitis C virus (HCV) infections are frequent^1^. In Taiwan, the disease is the leading cause of cancer mortality and affects 25/100,000 men and 10/100,000 women every year^2^. Liver transplantation has been shown to be a curative surgical management that also addresses underlying cirrhosis^3^, but hepatic resection remains the first option for patients with adequate reserve liver functionality as a result of limited organ availability^4^. Despite improvements in treatment strategies, HCC continues to present major challenges in management and its death rate remains high even after curative hepatectomy. Several tumor markers, such as alpha-fetoprotein (AFP), Lens culinaris agglutinin-reactive fraction of AFP (AFP-L3) and Des-gamma carboxyprothrombin (DCP), have been demonstrated to have prognostic value in HCC patients^5^, and their elevation reflects the progression of the disease. Yet the significance of these tumor markers is lower in patients with early stage HCC, and it could be challenging to employ these pretreatment tumor markers for forecasting the result of treatable early stage HCC.

Liver fibrosis is a common risk factor for the development of HCC, and the association of liver fibrosis with prognosis after curative hepatectomy has been demonstrated in previous studies^6,7^. Furthermore, approximately 80% of HCC patients are associated with hepatitis B or hepatitis C viral infections and varying degrees of liver fibrosis and cirrhosis^3^. Thus, assessment of the severity of liver fibrosis is essential when evaluating the prognosis of patients with HCC liver fibrosis, but it is often associated with significant costs and a risk of bleeding. Non-invasive and reliable methods are therefore urgently needed, and several modalities have been investigated. Serum Wisteria floribunda agglutinin-positive Mac-2-binding protein (WFA+-M2BP) level has been recognized as a novel biomarker for liver fibrosis and has been determined to be superior to other conventional markers in discrimination capability with respect to liver fibrosis^8–11^. Furthermore, WFA+-M2BP has been used as a predictor of HCC development in chronic hepatitis patients^12^. However, the association between it and prognosis in early stage HCC patients remains unknown. Since HBV and HCV infections are the most important causes of HCC and account for 80% of HCC cases globally^3^, we aim to evaluate the clinical significance of pretreatment serum WFA+-M2BP level as it relates to liver fibrosis, focusing on patients with HBV- and HCV- related early-stage hepatocellular carcinoma (ESHCC) who undergo liver resection. This information can be crucial in improving surgical options with respect to risks and potential benefits in this setting.

## Results

### Clinical characteristics

Six hundred and ten patients were identified and 198 were removed after application of the exclusion criteria; this left a final cohort of 412 patients. The clinical profile of the patient cohort is shown in Table 1. The mean age was 63.1 years, and there was a male predominance (74.8%). The number of cases of HBV HCC and HCV HCC were 240 (58.3%) and 172 (41.7%), respectively. Fibrosis stage based on the METAVIR score was F0 for 5 cases (1.2%), F1 for 50 cases (12.1%), F2 for 116 cases (28.2%), F3 for 94 cases (22.8%) and F4 for 147 cases (35.7%). The median follow-up time was 4.33 years. Cancer-related death occurred in 117 (28.4%) patients, and 295 patients survived. Using the ROC curve, we determined the recommended cutoff value of WFA+-M2BP was 1.12 with a sensitivity of 78.6% and a specificity of 52.5%. The AUC value was 0.694 (95% CI: 0.647–0.738, p < 0.0001). We further conducted subgroup analyses on HBV HCC and HCV HCC cohorts to determine the optimal cut-off values in predicting the overall survival. The optimal cut-off values were 1.12 (sensitivity: 71.4%, specificity: 60.9%) and 2.26 (sensitivity: 62.3%, specificity: 72.1%) for the HBV and HCV groups, respectively (Table 2). With the cut-off value of 1.12, patients were stratified into a low WFA+-M2BP group (n = 175) and a high WFA+-M2BP group (n = 237). Table 1 compares the clinical and laboratory parameters of the two groups. The high WFA+-M2BP group was older and less frequently male and had a significantly higher proportion of patients with AFP > 20 ng/mL. It also had higher AST and ALT levels, and its platelet count and median follow-up time were lower.

**Table 1:** Clinical characteristics of the study’s patients (*n* = 412).

		WFA+-M2BP ≤ 1.12 n = 175 (%)	WFA+-M2BP > 1.12 n = 237 (%)	*p* value	Total number of patients n = 412 (%)
Age (years)		60.17 ± 11.58	65.18 ± 9.87	<0.001	63.05 ± 10.90
Gender	Male	146 (83.4)	162 (68.4)	<0.001	308 (74.8)
	Female	29 (16.6)	75 (31.96)		104 (25.2)
BMI (kg/m^2^)		24.31 ± 3.93	24.64 ± 3.50	0.373	24.50 ± 3.68
Underlying disease					
	HBV	125 (71.4)	115 (48.5)	<0.001	240 (58.3)
	HCV	50 (28.6)	122 (51.5)		172 (41.7)
Stage					
	I	80 (45.7)	81 (34.2)	0.018	161 (39.1)
	II	95 (54.3)	156 (65.8)		251 (60.9)
METAVIR Score	0	2 (1.1)	3 (1.3)	<0.001	5 (1.2)
	1	34 (19.4)	16 (6.8)		50 (12.1)
	2	64 (36.6)	52 (21.9)		116 (28.2)
	3	38 (21.7)	56 (23.6)		94 (22.8)
	4	37 (21.1)	110 (46.4)		147 (35.7)
**Fatty liver**				0.112	
	Mild (0-1)	155 (88.6)	223 (94.1)		378 (91.7)
	Moderate (2)	16 (9.1)	10 (4.2)		26 (6.3)
	Severe (3)	4 (2.3)	4 (1.7)		8 (1.9)
AST (U/L)		45.02 ± 49.72	63.91 ± 40.16	<0.001	55.89 ± 45.39
ALT (U/L)		44.81 ± 50.04	60.04 ± 42.76	0.001	53.57 ± 46.55
Alpha-fetoprotein (ng/mL)				0.036	
	20 ≤	111 (64.2)	120 (51.7)		231 (57.9)
	20–400	38 (22.0)	74 (31.9)		112 (27.7)
	>400	24 (13.9)	38 (16.4)		62 (15.3)
Bilirubin (mg/dL)		0.92 ± 1.50	0.87 ± 0.40	0.612	0.89 ± 1.02
PLT (×10^3^/µL)		176.43 ± 55.40	152.57 ± 66.50	0.001	162.70 ± 63.07
APTT (sec.)		32.41 ± 4.70	32.38 ± 4.36	0.959	32.39 ± 4.50
PT (sec.)		11.40 ± 1.16	11.47 ± 1.28	0.527	11.44 ± 1.23
INR		1.06 ± 0.11	1.15 ± 0.86	0.166	1.11 ± 0.66
Activity	0	67 (38.3)	64 (27.0)	0.015	131 (31.8)
	1	108 (61.7)	173 (73.0)		281 (68.2)
WFA+-M2BP		0.76 ± 0.23	2.85 ± 2.23	<0.001	1.96 ± 1.99
Follow-up time	Median (SD)	4.64 (2.09)	4.15 (2.27)	0.041	4.33 (2.21)

**Table 2:** Serum WFA^+^-M2BP performance in estimation of overall survival.

	Cut-off value	AUC	95% CI	Sensitivity	Specificity	*p* value
**Total; n** = **412**						
WFA+-M2BP	1.12	0.694	0.647–0.738	78.63	52.54	<0.0001
**HBV; n** = **240**						
WFA+-M2BP	1.12	0.652	0.588–0.712	71.43	60.87	0.0006
**HCV; n** = **172**						
WFA+-M2BP	2.26	0.712	0.639–0.779	62.30	72.07	<0.0001

### Correlation between liver fibrosis and serum WFA+-M2BP level

Diagnostic test performance of WFA+-M2BP values in liver fibrosis is shown in Table 3. The AUC values in predicting fibrosis stages ≥F2, ≥F3, and ≥F4 were 0.670 (95% CI: 0.622–0.715, p < 0.0001), 0.697 (95% CI: 0.651–0.741, p < 0.0001), and 0.705 (95% CI: 0.658–0.749, p < 0.0001), respectively. Optimal cut-off values were 1.03 for ≥F2 (sensitivity: 67.2%, specificity: 61.8%), 1.90 for ≥F3 (sensitivity: 43.2%, specificity: 87.1%), and 2.45 for ≥F4 (sensitivity: 41.5%, specificity: 89.8%). In the subgroup analysis of 240 HBV HCC patients, AUCs were 0.637, 0.634, and 0.666 for ≥F2, ≥F3, and ≥F4, respectively. The optimal cutoff values that best predicted fibrosis stages ≥F2, ≥F3, and ≥F4 were 1.03, 1.32, and 1.03, respectively. Whereas in the analysis of the HCV HCC cohort, AUCs were 0.698, 0.792, and 0.801 for ≥F2, ≥F3, and ≥F4, respectively.

**Table 3:** Serum WFA^+^-M2BP performance in estimation of liver fibrosis stage in chronic hepatitis B/C patients.

	Cut-off value	AUC	95% CI	Sensitivity	Specificity	*p* value
**Total; n** = **412**						
F0-F1 vs F2-F4	1.03	0.670	0.622–0.715	67.23	61.82	<0.0001
F0-F2 vs F3-F4	1.90	0.697	0.651–0.741	43.15	87.13	<0.0001
F0-F3 vs F4	2.45	0.705	0.658–0.749	41.50	89.81	<0.0001
**HBV; n** = **240**						
F0-F1 vs F2-F4	1.03	0.637	0.573–0.698	58.71	66.67	0.0053
F0-F2 vs F3-F4	1.32	0.634	0.569–0.695	47.01	75.47	0.0002
F0-F3 vs F4	1.03	0.666	0.603–0.725	71.26	54.90	<0.0001
**HCV; n** = **172**						
F0-F1 vs F2-F4	2.21	0.698	0.624–0.766	44.87	93.75	0.0007
F0-F2 vs F3-F4	2.21	0.792	0.723–0.850	59.81	89.23	<0.0001
F0-F3 vs F4	2.45	0.801	0.734–0.858	66.67	83.93	<0.0001

### Prognostic value of WFA+-M2BP levels on overall survival after hepatic resection

**Figure 1:**
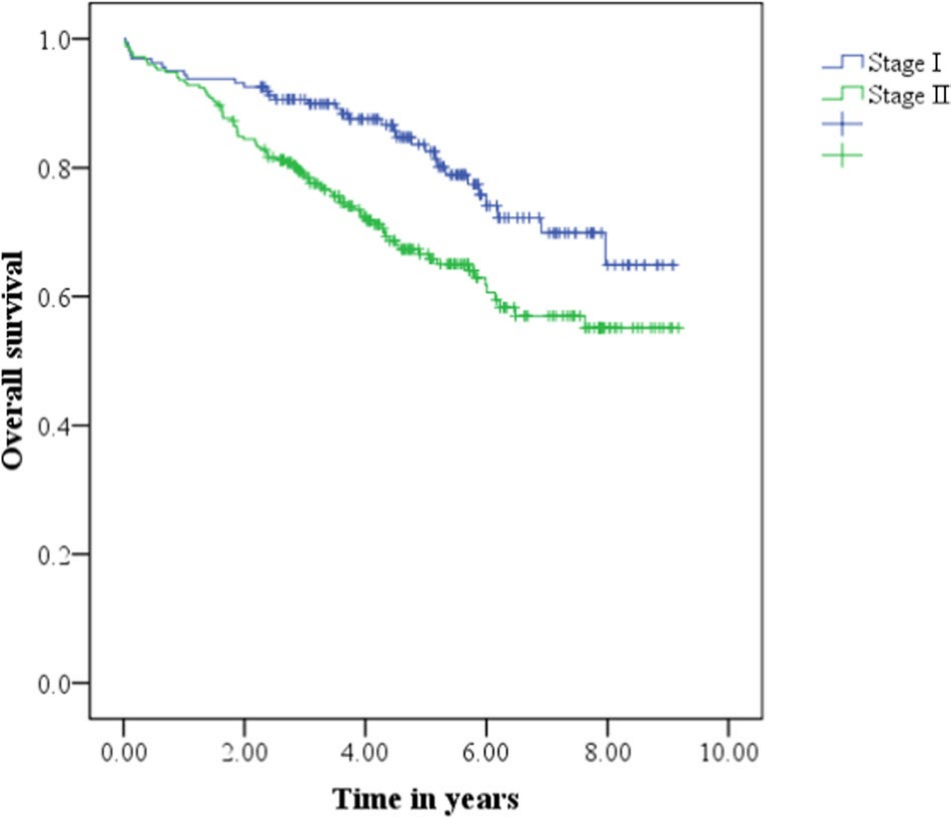
Kaplan-Meier curve demonstrating the overall survival rates after curative hepatic resection. Overall survival curve stratified by TNM stage. Survival rates were significantly higher in patients with stage I disease (blue line) than those with stage II disease (green line). Log rank p value = 0.002.

**Figure 2:**
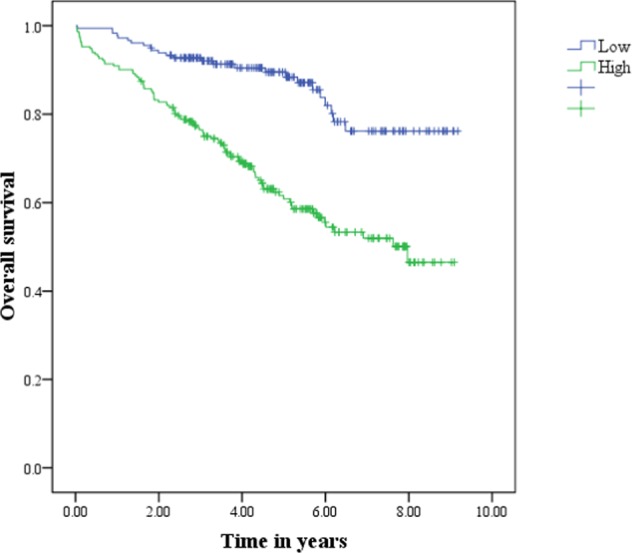
Kaplan-Meier curve demonstrating the overall survival rates after curative hepatic resection. Overall survival curve stratified by pretreatment WFA+-M2BP levels. Survival rates were significantly higher in patients with a WFA+−M2BP level ≤ 1.12 (blue line) than those with a WFA+−M2BP level> 1.12 (green line), Log rank *p* value <0.001.

Kaplan-Meier (KM) survival analysis showed a significant difference in the probability of survival across the different TNM stages (Fig. 1). The estimated 3- and 5-year overall survival rates after liver resection in patients with TNM stage I were 91% and 83% compared to 79% and 67% in patients with TNM stage II. Kaplan-Meier survival analysis showed the overall survival rates were higher in the TNM stage I group than in the TNM stage II group (p = 0.002). The association between serum WFA+-M2BP levels and overall survival is shown in Fig. 2. The estimated 3- and 5-year overall survival rates after liver resection in patients with WFA+-M2BP levels ≤ 1.12 were 92% and 90% compared to 76% and 61% in patients with WFA+-M2BP levels >1.12. KM analysis demonstrated a significantly lower survival rate in patients with WFA+-M2BP levels >1.12 (p < 0.001). The estimated median survival time and the 3- and 5-year survival rates based on TNM stage and serum WFA+-M2BP level are summarized in Table 4. We next performed univariate and multivariate analyses to determine factors associated with cancer-related death. In the univariate analysis, variables associated with cancer-related death were age (HR: 1.029, p = 0.003), fibrosis grade (HR: 1.209, p = 0.015), pre-operative serum AFP level (HR: 1.308, p = 0.026), tumor stage (HR: 1.854, p = 0.003) and pre-operative serum WFA+-M2BP level (HR: 3.178, p < 0.001). Risk factors for cancer-related death were further analyzed in a multivariate regression model to identify the most important factors (Table 5). Based on the multivariate regression model, only age (HR: 1.022,p = 0.031), fibrosis grade (HR: 1.217, p = 0.042), cancer stage (HR: 1.726, p = 0.01) and WFA+-M2BP level (HR: 2.290, p = 0.001) were independently associated with overall survival in patients with ESHCC. During the analysis of survival prediction, serum WFA+-M2BP level had a higher log-likelihood and a lower AIC value compared to TNM stage (WFA+-M2BP: log likelihood = −639, AIC = 1280; TNM stage: log likelihood = −648, AIC = 1298).

**Table 4:** Overall survival at 3 years and 5 years stratified by stage and serum WFA+-M2BP level (n = 412).

	Patients	MST (yrs)	95% CI	3-yr OS (%)	5-yr OS (%)	*p* value
WFA+-M2BP ≤ 1.12	180	8.005	7.586–8.424	92	90	<0.001
WFA+-M2BP > 1.12	232	6.127	5.666–6.587	76	61	
Stage I	161	7.477	7.000–7.954	91	83	0.002
Stage II	251	6.563	6.117–7.009	79	67

**Table 5:** Univariate and multivariate analyses of factors associated with post-operative overall survival in stage I and stage II hepatocellular (HCC) patients after curative hepatic resection (n = 412).

	Overall survival					
	Univariate analysis			Multivariate analysis		
	**HR**	95% CI	*p* value	HR	95% CI	*p* value
Age	1.029	1.010–1.048	0.003	1.022	1.002–1.043	0.031
METAVIR	1.209	1.037–1.410	0.015	1.217	1.007–1.470	0.042
AST	1.001	0.998–1.005	0.467	—	—	—
ALT	1.000	0.997–1.004	0.867	—	—	—
AFP	1.308	1.033–1.656	0.026	1.172	0.920–1.493	0.199
Stage (I vs II)	1.854	1.239–2.775	0.003	1.726	1.142–2.609	0.010
WFA+-M2BP ( ≤ 1.12 vs >1.12)	3.178	2.042–4.946	<0.001	2.290	1.433–3.661	0.001

## Discussion

The present analysis of serum WFA+-M2BP covered HCC of different etiologies: HBV and HCV. Overall, the diagnostic performance in distinguishing between the different stages of fibrosis was found to be unsatisfactory in the current study (AUC: ≥F2 = 0.670, ≥F3 = 0.697, and ≥F4 = 0.705). However, as the severity of fibrosis increased, specificity climbed from 61.8% to 89.8%, suggesting that WFA+-M2BP could be employed to identify advanced fibrosis or cirrhosis. In the subgroup analysis, the diagnostic accuracy of WFA+-M2BP was inferior in the HBV HCC cohort when compared to the HCV HCC cohort. This suggested fibrosis progression may vary according to the etiology of the liver disease. HBV-related fibrosis was reported to be associated with large regenerative nodules with thin septa; moreover, the background fibrosis often fluctuates during the course of the disease^13,14^. In contrast, HCV-associated fibrosis has a more insidious course and a slower fibrosis progression and is correlated with small nodules. Therefore, we should be aware that the optimal cutoff values of WFA+-M2BP for fibrosis vary according to the etiology of the chronic liver disease. A low specificity of 52.5% was reported for the WFA+-M2BP cutoff value of 1.12 (Table 2) in predicting the overall survival. However, when we conducted subgroup analyses, the optimal cut-off values were 1.12 (sensitivity: 71.4%, specificity: 60.9%) and 2.26 (sensitivity: 62.3%, specificity: 72.1%) for the HBV HCC and HCV HCC groups, respectively. These increases in specificity suggested that the WFA+-M2BP cutoff value for predicting the overall survival may differ depending on the etiology, and the low specificity reported in the first place may be a result of the diversity of the studied subjects included in the present investigation.

The proportion of F4 in patients with WFA+-M2BP > 1.12 was significantly higher than that in patients with WFA+-M2BP ≤ 1.12 (46.4% vs. 21.1%, p < 0.001). The results were reasonable as WFA+-M2BP has been designated as a meaningful predictor for liver fibrosis in various etiologies of chronic liver disease^8,9,15^. The elevation of serum WFA+-M2BP levels reflects the production of glycoproteins by hepatocytes during the process of liver fibrosis or inflammation. Hepatic stellate cells (HSCs) are the main cell type responsible for WFA+-M2BP secretion, and the interaction of M2BP with Mac-2 expressing Kupffer cells would further modulate the activation of HSCs^16^. Activated HSCs then markedly contribute to liver fibrosis and portal hypertension, and they also play an important part in liver carcinogenesis and metastasis^17^. Therefore, recent studies have suggested the importance of WFA+-M2BP in the development of HCC as a modulator of liver fibrosis and tumor cell microenvironment^12,18^. Serum WFA+-M2BP levels were notably higher in patients with early stage steatosis (Table 1, n = 223; 59.0%) in our results, and this suggested that WFA+-M2BP could be a potential marker of early stage liver injury as previously reported. Nishikawa *et al*. showed a significant correlation between CRP concentration and liver inflammatory activity in their earlier study of patients with autoimmune hepatitis^15^. Ura *et al*. also reported that the WFA+-M2BP level was positively correlated with ALT, AFP and the activity grade, and their data suggested the serum WFA+-M2BP level may be influenced not only by fibrosis but also by liver inflammation since ALT and AFP elevations reflect liver inflammation or regeneration^19^.

HCC is different from other solid tumors because the presence of chronic liver disease and cirrhosis influence overall survival and treatment outcome. Many staging systems have been established, but there is no consensus on the best classification to employ. The TNM system proved to be the best for prognostic stratification and prognostic prediction in an earlier study of 234 HCC patients who underwent curative resection in China^20^. However, the Okuda, Barcelona and Cancer of Liver Italian Program (CLIP) staging systems were more effective for predicting outcomes in patients who underwent non-surgical therapy^21^. When the tumors were stratified according to different TNM stages in our cohort, there was a statistically significant difference in overall survival between stage I and stage II. In addition, multivariate analysis showed that patients with stage II disease had a 72.6% higher risk (HR: 1.726, 95% CI = 1.142–2.609) for mortality compared with those with stage I disease. This suggests that early diagnosis and treatment are essential to enhance survival in HCC patients. Despite its ease of use and convenience in the surgical setting, TNM staging may not be sufficient to stage HCC since the system does not consider residual liver function, which is often crucial for the prognosis of HCC patients^22,23^. In the comparative analysis of the TNM staging system and WFA+-M2BP serum levels conducted by the current study, both prognostic tools were individually important in the prediction of overall survival among the analyzed population and displayed comparative predictive performance.

In the past two decades, the use of interferon-based antiviral therapy has resulted in the benefit of reducing development of cirrhosis and HCC for patients with chronic hepatitis C (CHC) who achieved sustained virological response (SVR). The addition of direct-acting antivirals (DAAs) has further improved the SVR rates of antiviral therapy to more than 90%, even for patients with cirrhosis^24^. A recent meta-analysis showed that CHC individuals with SVR have a considerably reduced risk for HCC incidence and liver-related mortality as compared to treatment failure^25^. Several studies have focused on evaluating treatment efficacy for the association between serum WFA+-M2BP levels and prognostic performance after achieving SVR. Sato *et al*. showed a significantly higher incidence of HCC development after SVR in patients with high serum WFA+-M2BP (p < 0.001)^26^. Furthermore, Hasegawa *et al*. reported that the absence of hepatocellular carcinoma (p = 0.0008), WFA+-M2BP < 6.15 COI (p = 0.013), achievement of sustained virological response (p < 0.0001) and des-carboxy prothrombin <41 mAU/mL (p = 0.0018) were significantly favorable factors associated with survival in HCV-related compensated liver cirrhosis^27^.

In the present analysis, the patients with WFA+-M2BP > 1.12 were older in age with higher AST and ALT levels and lower platelet counts. Previous reports demonstrated that older HCC patients are more likely to present with cirrhosis than younger patients, and this might be attributed to aged patients having a longer liver disease duration and subsequently a higher risk of cirrhosis development^28,29^. Elevated AST and ALT levels in patients with WFA+-M2BP levels >1.12 reflect the current liver damage and acute liver inflammation. A low platelet count has been a common finding associated with HCC patients, which may be explained by the pooling of platelets in an enlarged spleen secondary to cirrhosis-induced portal hypertension^30^. Besides liver function tests, tumor progression is another important aspect to consider in HCC patients with regard to prognosis. Recent advances in imaging modality, such as multidetector-row CT and MRI, along with the development of contrast media have led to early detection of hepatic nodules, including small HCC tumors in the context of cirrhosis. Several tumor markers, including AFP, AFP-L3 and DCP, have been established specifically for disease management in patients with HCC^3,5,31,32^. The elevation of these markers often takes place when the size and number of HCC lesions increase or an invasion of the portal vein occurs^31–35^, thus reflecting HCC progression. But it would be difficult to employ serum tumor marker levels to forecast outcomes before curative treatments as their levels could be low in patients with early-stage HCC. A few studies compared the diagnostic performances of conventional biomarkers with WFA+-M2BP in detecting ESHCC, and the results have been inconsistent. Chuaypen *et al*. reported on 150 pairs of matched cases and found that WFA+-M2BP was superior to AFP in differentiating ESHCC from advanced fibrosis without HCC^18^. Furthermore, previous data from Yamasaki *et al*. also indicated a better diagnostic performance of WFA+-M2BP than that of AFP for predicting HCC development in long-term follow-up of patients with chronic HCC infections^36^. In contrast, the study by Hasegawa *et al*. demonstrated that hyaluronic acid had a higher prognostic performance than WFA+-M2BP and other biomarkers in predicting overall survival among HCV-related HCC patients^27^.

Another meaningful result from our study is that we identified WFA+-M2BP level before curative hepatectomy as an independent predictor of mortality in patients with early HCC; this was done through univariate and multivariate analyses. In addition, the significantly different survival rates among the two groups of WFA+-M2BP levels (≤1.12 and t>1.12) supported a significant association between the WFA+-M2BP values and the overall survival of early stage HCC patients. Unlike tumor markers mentioned earlier, serum WFA+-M2BP level was not correlated with tumor stage and size, hence it would be a better biomarker of disease progression and overall prognosis.

Furthermore, our analysis revealed that increasing age is another independent factor for poor overall survival. There are a few studies assessing the influence of age on post-resection outcomes in HCC, and the results have been inconsistent. Kaibori *et al*.^37^ reported on 12,587 patients in a Japanese nationwide study that indicated that elderly patients had significantly worse overall survival after hepatectomy than young patients. In contrast, Su *et al*.^38^ and Tan *et al*.^39^ both suggested older patients with adequate remnant liver reserves and good liver functionality should be considered for curative resection since age was not determined as an independent predictor for overall survival postoperatively. Liver functionality is a crucial factor affecting the prognosis of patients with HCC. However, parameters like bilirubin and albumin were not found to be significantly associated with overall survival in our univariate analysis. This might be attributed to our study design, which targeted on those patients with good liver functionality and mild fibrosis who could undergo curative resection in the first place.

There are a few limitations to our study. First, this was a retrospective, single institution study, although studied patients were followed prospectively. Second, the current study was limited to early stage HCC patients, and the predictive and prognostic value of WFA+-M2BP in patients with more advanced HCC remained to be investigated. Furthermore, the association between the changes in WFA+-M2BP and fibrosis could not be measured as repeated liver biopsies were not performed during the follow-up period. Therefore, further prospective studies with greater sample sizes are necessary and our results should be interpreted with caution. Nonetheless, in the current study we demonstrated that serum WFA+-M2BP level at diagnosis before surgery can be helpful for predicting the overall survival. In conclusion, our data suggest that evaluating WFA+-M2BP before curative surgery is an easy, noninvasive and useful apparatus not only for predicting the fibrosis stage in early-stage HCC, but also for estimating prognosis.

## Methods

### Study

This study was approved by the institutional review board of Changhua Christian Hospital, Changhua, Taiwan (No. 120611 and No. 150815), and written informed consent for the use of their clinical specimens was obtained from all participants. Our institutional prospectively maintained database was reviewed to identify pathologically confirmed HCC patients who underwent hepatectomy with curative intent between April 2009 and January 2016. The diagnosis of HCC was based on typical imaging with positive enhancement in the arterial phase followed by contrast washout in the delayed phase or histopathology by biopsy in cases with atypical enhancing patterns as reported in our previous study^40^. Exclusion criteria were stage III/IV HCC, lost to follow-up, concurrent presence of other types of cancer, non-cancer related death and non-B, non-C HCC. Disease stage was classified on the basis of the 2010 staging system of the American Joint Committee of Cancer (AJCC) TNM criteria. Using the METAVIR score system, the severity of liver fibrosis was determined histopathologically in resected non-cancerous liver tissue on a scale of 0 to 4^41^. In short, tissue samples were fixed in buffered formalin and embedded in paraffin. Samples were subjected to standard techniques for hematoxylin and eosin and Masson trichrome staining. Blood samples were collected during the outpatient preoperative evaluation or on the day of admission. Clinical parameters of interest included age, sex, body mass index (BMI), liver function test and etiologic factors predisposing toward HCC. All study procedures were performed in accordance with relevant guidelines and regulations.

### Measurement of WFA+-M2BP

Pretreatment serum levels of WFA+-M2BP were quantified by a lectin-Ab sandwich immunoassay using a commercially available kit (HISCL-800; sysmex Co., Kobe, Japan)^8,9^, followed by adjustments during the automatic assay.

### End points and statistical analysis

Time to event was defined as the interval from the definite surgery to the date of cancer related death. Clinicopathological characteristics were compared by the Mann-Whitney *U* test for medians and the chi-square test for proportions. Risk factors influencing overall survival were analyzed by univariate and multivariate methods using the Cox proportional hazard regression model. A hazard ratio (HR) and its 95% confidence interval (CI) were determined for each independent risk factor. Receiver operating characteristic (ROC) curve analysis was employed to determine the optimal cut-off value for WFA+-M2BP based on the Youden index^42^. The diagnostic performance of the cut-off value was expressed in terms of the diagnostic sensitivity, specificity, and area under the ROC curve (AUC). Kaplan-Meier (KM) survival curves were generated to compare the survival outcomes^43^, and a two-sided log rank test was used to test the differences between the survival experiences regarding different cancer stages (I and II) and WFA+-M2BP levels^44^. Univariate and multivariate analyses of factors related to overall survival were performed using the Cox proportional hazard model. Variables that reached statistical significance were further included in the multivariate analysis. The prognostic performance among the studied population of a stage system or scoring system was statistically assessed using the Akaike Information Criterion (AIC) and log likelihood. The best model for predicting overall survival would be a model with a lower AIC value and a higher log likelihood value. Statistical analyses were performed using MedCalc for Windows (version 18.11.6, MedCalc Software bvba, Ostend, Belgium) and the statistical package SPSS for Windows (version 20.0.0; SPSS, Chicago, IL, USA); a significance level of 5% was used throughout.
